# The Association Between Racial Microaggressions and Stereotypes and Self-Rated Mental Health in Asian Women

**DOI:** 10.3390/ijerph22121869

**Published:** 2025-12-15

**Authors:** Yvette C. Cozier, Bhavana Ganduri, Michael Tang, Yifan Xie, Uyen-sa D. T. Nguyen, Hyeouk Chris Hahm

**Affiliations:** 1 Department of Epidemiology, Boston University School of Public Health, Boston, MA 02118, USA; bhavanag@bu.edu (B.G.); mtang11@bu.edu (M.T.); 2 Department of Human Behavior, Research, and Policy, Boston University School of Social Work, Boston, MA 02215, USA; xyifan@bu.edu; 3 Department of Population & Community Health, College of Public Health, University of North Texas Health Sciences Center, Fort Worth, TX 76107, USA; uyen-sa.nguyen@unthsc.edu

**Keywords:** Asian, Asian American, microaggression, model minority, racism, discrimination

## Abstract

The impacts of microaggressions and the Model Minority Myth on self-rated mental health among Asian American (AA) women are unclear. A total of 152 AA women completed an online questionnaire including select items from the Racial and Ethnic Microaggressions Scale (REMS) and the Internalization of the Model Minority Myth Measure (IM-4). Logistic regression was used to estimate associations (odds ratios and 95% confidence intervals) between the REMS and IM-4 with fair/poor mental health. Experiencing frequent microaggressions was significantly and consistently associated with fair/poor mental health, particularly those experiences involving exclusion or dismissal during interpersonal or professional interactions. Women who reported more microaggressions (>10), compared to those who reported fewer (<5), had more than twice the odds of fair/poor mental health (OR: 2.70, CI: 1.12, 6.49). For the IM-4, compared to those who were undecided, women who agreed with statements such as “Asian Americans have stronger work ethics” had lower odds of poor or fair/poor mental health: (OR: 0.39, CI: 0.15, 0.99) than those who were undecided. Gaining a greater understanding and acknowledgement of the impacts of subtle microaggressions and stereotyping, including internalization of stereotypes, is necessary to support psychological wellbeing and design effective mental health interventions for Asian American women.

## 1. Introduction

In the United States (US), Asians comprise the fastest-growing racial or ethnic group [1]. Between 2000 and 2019, the US Asian population grew from approximately 11 million to 19 million, and it is projected to rise to 36 million by 2060 [1]. There is vast diversity among Asian Americans (AA) in the form of distinct ethnic groups from different countries of origin including China, Korea, Japan, the Philippines, Vietnam, and India [2]. There are also substantial intra-racial differences according to background (e.g., being multiracial), migration, acculturation, language, socioeconomic status, and residence [3].

Asian Americans are often portrayed as a successful group in terms of education achievement and economically [4]. Behind this image of success, however, AAs experience high rates of mental disorders including depression, anxiety, and PTSD [5]. Chronic exposure to race-related psychosocial stressors has been associated with poor physical [6,7,8,9] and mental health in marginalized populations, including suicidal behaviors and substance abuse [10,11,12,13]. Specifically, racial microaggressions and stereotypes have been linked to negative coping mechanisms, risk factors for chronic illnesses, and negative psychological symptomatology (i.e., depression, low self-esteem, refusal to seek help, suicidal ideation) [14,15]. The overarching goal of this study is to explore the relationship between chronic psychosocial stress and mental health in AA women.

### 1.1. Historic and Cultural Context

The AA experience extends to the middle 1800s when immigrants, mostly men from China, Japan, and the Philippines, filled many low-paying industrial jobs including mining, railroad construction, and farming. In the late 19th and early 20th centuries, Koreans and South Asians began to arrive in the US, followed by refugees from Vietnam in the 1970s [16]. Over the course of their history, Asian immigrants and AAs have faced structural and interpersonal forms of racism including the Chinese Exclusion Act of 1882 [17], the Johnson-Reed Act of 1924 [18], and Executive Order 9066 in 1942 [19]. Overt forms of racism include the Chinatown massacre of 1871 [20], the death of Vincent Chin [21], and most recently, anti-Asian sentiment in light of the COVID-19 pandemic [22,23,24]. In the years prior to the pandemic, anti-AA racial discrimination was more covert, often in the form of pervasive microaggressions and stereotypes [25].

### 1.2. Microaggressions and Stereotypes Versus Overt Racism

Unlike overt racial discrimination, racial microaggressions are frequent, subtle, implicit acts of discrimination against members of marginalized groups [14,15,26,27]. They consist of verbal and non-verbal messages of belittlement, including well-intentioned, yet derogatory verbal statements (i.e., backhanded compliments) [14,26]. Racial microaggressions are challenging to address due to their intangible nature. Indeed, the term “micro” refers to the fact that the aggression itself may seem harmless or insignificant to those not targeted [28]. The recipients, however, do not interpret these experiences as being trivial [26,29], and are emotionally and ultimately, physiologically triggered [30]. The subtlety of these actions results in the target experiencing “attributional ambiguity”, or being unsure of whether the actions were driven by racism, followed by uncertainty whether the action was misinterpreted and/or intentional [28,31]. Studies conducted among US undergraduate college students of color (including AAs) have shown that those reporting more experiences of microaggressions also reported greater psychological distress, anxiety and binge alcohol drinking [31,32]. Asian medical students experiencing one or more race-related microaggressions during their training subsequently reported feelings of uncertainty about the microaggression itself, anger, frustration, fear of consequences for reporting the experience, isolation, and burnout [33].

A stereotype is defined as “a fixed, over-generalized belief about a particular group or class of people” [34]. Racial stereotypes promote internalized racism, or the adoption of “bias and oppression towards one’s heritage group” [35]. The “model minority myth” (MMM) is one such stereotype applied to AAs. Initially introduced in the 1960s as an attempt to undermine the Civil Rights movement, the MMM is the belief that AAs are universally more successful academically, economically, and socially compared to other racial minority groups [36], and that racism can be overcome simply through hard work and achieving the American Dream. This stereotype aligns with a “color-blind” ideology by elevating AAs as an intermediary group between White and Black Americans to exemplify what other minoritized groups could achieve through hard work and perseverance, shifting the focus away from racism [14,37,38]. Whether seemingly positive (e.g., “all Asians are good at math”) or negative (e.g., “all Asians lack leadership skills”), stereotypes strip individuals of their distinctiveness, create scapegoats, and reinforce biases [39]. In particular, they homogenize the experiences of people of Asian descent [40], rendering invisible the experiences of less educated, working class, and poor Asian individuals and families [41].

A growing body of research among AAs has explored the association between internalized racism and adverse mental health outcomes, resulting in mixed and conflicting findings. Gupta et al. [42] found that AAs endorsing positive stereotypes reported higher levels of psychological distress and more resistance toward seeking help. Similarly, AA students attending a predominantly AA high school experienced increased depression and anxiety related to endorsement of positive stereotypes regarding upward mobility (e.g., “Asian Americans are less likely to experience racism”) [43]. In contrast, Kiang et al. [44] found that AA high school students who were aware of being perceived as a model minority demonstrated increased self-esteem and positive relationships with others. Chang and colleagues also observed an inverse association between internalization of the MMM and depressive symptoms among AA college students [45], while other studies found no significant risk of depression or depressive symptoms among AA women reporting high levels of internalized racism [46,47,48].

Both microaggressions and the MMM are complex, ambiguous phenomena that may be interpreted differently by each individual. For microaggressions, there is uncertainty on whether one is being complimented or judged on the basis of their race or ethnicity [32]. Similarly, individual appraisal of the MMM may differ depending on whether an individual sees themselves as conforming to or rejecting the stereotype. A survey found that approximately 40% of Asian adults felt the term “model minority” to describe Asians was negative; 17% said it was positive, and 40% felt it was neither good nor bad, or unsure [49]. Thus, those who feel that they “fit” the stereotype may perceive the MMM as a positive affirmation that they must strive to achieve. Alternatively, those who do not fit the stereotype may view the MMM as an imposition of unrealistic or unfair expectations [4]. Finally, the uncertainty and divergent personal experiences related to the MMM may also lead some individuals to feel either neutral or “undecided” in their appraisal. For example, a Pew survey respondent noted feeling placed in a “weird middle ground” between stereotype expectations of unrestricted success and the personal reality that “All of us are not by any means” high achieving and prosperous [49]. Ultimately, navigating such ambiguity may be stressful and may impact mental health [26] regardless of appraisal category.

### 1.3. Microaggressions, Stereotypes, and the Wellbeing of Asian Women

Compared to AA men, AA women experience distinct forms of oppression reflecting the intersection of gender and race. Historically, gendered racial stereotypes have fetishized AA women as domestic, submissive, and sexually subservient objects, making them increasingly vulnerable to sexual and physical violence [26,50]. For example, AA women were more than twice as likely to report a COVID-19-related hate incident than AA men, and the victims of the 2021 Atlanta spa shootings were mostly women [51]. Empirical studies exploring discrimination, however, have typically focused on race, overlooking the gendered aspects of such experiences [50]. Studies suggest that such gendered racial microaggressions are linked to poor mental health among AA women, including depressive symptoms and suicide ideation [50,52]. Thus, it is important to explore how microaggressions and stereotypes impact AA women.

In the current analysis, we explored the role of microaggressions and the MMM in relation to self-rated mental health, an indicator of mental well-being and social functioning [53], in a cohort of AA women. We hypothesize that frequently experiencing microaggressions is associated with poor self-rated mental health. We also hypothesize that compared to those who express ambiguity (undecided), greater endorsement (agreement) of the MMM is associated with better self-rated mental health, while disagreement is associated with poorer self-rated mental health.

## 2. Materials and Methods

### 2.1. Study Population

The current analysis involves participants from the Epidemiology and Epigenetics of Asian Women’s Action for Resilience and Empowerment (Epi AWARE) study whose methods have been described elsewhere [23]. Briefly, Epi AWARE participants consisted of two groups. The first group of women were recruited from the AWARE (Asian Women’s Action for Resilience and Empowerment) Study [54], an NIH-funded randomized intervention regarding Asian women’s mental and sexual health. AWARE participants with an email address on file as of November 2019 were invited to join the Epi AWARE study by completing a consent form and the Wave 1 online questionnaire. The second group of women was recruited locally, with AA women learning about the study through printed flyers placed on college campuses and in community centers, advertisements on social media (Facebook, Instagram, Twitter (now X)), and through word of mouth. Interested women contacted the study and were subsequently emailed a letter containing a link to the Epi AWARE consent form and Wave 1 online questionnaire. A total of 183 women who self-identified as Asian or Asian-American enrolled by completing an online questionnaire between December 2019 and September 2022 [8,23]. Participants were between 18 and 59 years of age (mean age = 26 years), mostly resided in the Northeastern US, and were primarily of Chinese, Korean, and Vietnamese ancestry. All participants provided informed consent. The study protocol was approved by the Boston University Medical Center Institutional Review Board.

### 2.2. Microaggressions

The Racial and Ethnic Microaggressions Scale (REMS) [15] consists of 45 self-report items across six dimensions. Due to space limitations, our questionnaire utilized a total of 26 items covering all domains from the original instrument (Cronbach’s α = 0.829): Assumptions of Inferiority (4 items, α = 0.498; e.g., “*someone told me I was* “*articulate” after assuming I wouldn’t be*”); Second-class Citizen and Assumptions of Criminality (5 items, α = 0.592; e.g., “*someone avoided sitting next to me in a public space because of my race*”); Microinvalidations (6 items, α = 0.843; e.g., “*I was told I should not complain about race*”); Exoticization (4 items, α = 0.556; e.g., “*someone wanted to date me only because of my race*”); Environmental Microaggressions (3 items, α = 0.491; e.g., “*I observed that people of my race were the CEOs of major corporations*”); and Workplace and School Microaggressions (4 items, α = 0.846; e.g., “*I was ignored at school or at work because of my race*”). The REMS has shown high validity and reliability among minoritized populations including Asian Americans [15]. Our questionnaire utilized a total of 26 items from the original instrument to which participants could respond with a yes or no answer to events they experienced at least once within the past 6 months. We created a summary score representing the number of affirmative responses (range: 0–26). Higher scores represented greater exposure to microaggressions.

### 2.3. The Model Minority Myth

The Internalization of the Model Minority Myth Measure (IM-4) [36] is a 15-item self-report scale which has been validated among AA college students [55] and adolescents [56] and has demonstrated internal reliability and stability over two weeks [36]. Due to space limitations, our questionnaire contained 11 items across 2 dimensions (Cronbach’s α = 0.819): Achievement Orientation (IM4-AO) (7 items, α = 0.855) (e.g., “*Asian Americans have higher grade point averages in school because they work harder*”) and Unrestricted Mobility (IM4-UM) (4 items, α = 0.728) (e.g., “*Asian Americans are less likely to experience racial prejudice than other groups*”). Response options consisted of a 7-point Likert scale: strongly disagree (1), disagree (2), more or less disagree (3), undecided (4), more or less agree (5), agree (6), and strongly agree (7). We explored each individual question by collapsing responses into three categories: disagree (strongly disagree, disagree, more or less disagree), undecided, and agree (more or less agree, agree, and strongly agree). We also created an overall score variable by averaging the responses of the 11 questions, which were further divided into approximate tertiles. Higher scores represent greater agreement with the MMM.

### 2.4. Self-Rated Mental Health

Participants were asked the following question [53]: “In general, how would you rate your mental health?” Response options consisted of a 5-point Likert scale: excellent, very good, good, fair, and poor. This single-item measure is widely recognized as an indicator of population-level mental well-being and social functioning and is strongly associated with other mental health measures including the Center for Epidemiologic Studies Depression Scale (CES-D), Geriatric Depression Scale-Short Form (GDS-SF), and Patient Health Questionnaire-9 (PHQ-9) [53]. For ease of interpretation and to accommodate the limited size of our sample, responses were dichotomized into “excellent/very good/good” and “fair/poor”.

### 2.5. Covariates

We selected covariates based on previous literature and previous analyses within our cohort. Participants were asked to provide their age (years), highest level of educational attainment (years), and nativity (U.S. born, non-U.S. born). To account for Asian diversity [2,3], participants were asked to report their ethnicity which was further categorized according to geographic region (South East Asian, South Asian, East Asian). Finally, participants reported whether they had ever been diagnosed with depression treated with medication.

### 2.6. Statistical Analysis

The sample for this analysis consisted of the 152 participants with complete exposure (REMS and IM-4) and outcome (self-rated mental health) data. For categorical and ordinal variables, we calculated frequencies and percentages using chi-square tests, and we estimated means and standard deviations for continuous variables using t-tests. The REMS and IM-4 were each analyzed according to their individual component questions and as summary variables. Each REMS question was modeled as a dichotomous variable (yes vs. no (reference category)) while the REMS summary variable was modeled as an ordinal variable based on the number of affirmative responses (≤5 (reference category), 6–10, >10). The individual IM-4 questions were modeled as ordinal variables (agree, undecided (reference category), disagree). We attempted to further divide the averaged IM-4 score into tertiles, but due to tied values, we could not determine exact cut-points; we instead categorized them into “approximate tertiles” designated as 1 (low) (reference category), 2, and 3 (high) categories. The self-rated mental health outcome was modeled as a binary variable (“excellent/very good/good” (reference category), “fair/poor”). Logistic regression was used to estimate odds ratios (OR)—an estimate of relative risk [57]—and 95% Confidence Intervals (CI) for the association(s) of measures of REMS and IM-4 variables with self-rated mental health. We conducted stepwise multivariable logistic regression and only retained variables that changed the OR estimate by 10% or more. Thus, our main model (model 2) adjusted for age (≤26, >26 years), and education (<16, ≥16 years). We also considered the interrelation between the REMS and IM-4 measures and simultaneously adjusted for each item (model 3). Specifically, model 3 for each REMS item and the REMS summary score contained an additional term for the IM-4 summary score. Likewise, model 3 for each IM-4 item and the IM-4 summary score contained an additional term for the REMS summary score. Finally, we explored the overall REMS and IM-4 scores within strata of age (≤26, >26 years) and timing of questionnaire completion pre-pandemic (before 20 March 2020) and during the pandemic (“pandemic”) (on/after March 2020).

## 3. Results

The mean age of Epi AWARE participants was 28.7 years (SD: 9.3) (range: 18–59 years) (Table 1). Approximately 80% had earned a college (4-year) degree or higher, and two-thirds (64.5%) were born in the US. Over 70% reported East Asian (China, S. Korea, Japan) ethnic geography, and 20% reported Southeast Asian (Philippines, Vietnam) ethnic geography. Approximately 24% reported a diagnosis of depression treated with medication. Women who reported fair or poor mental health were, on average, younger (26.3 years vs. 30.2 years) and more likely to be U.S.-born (80% vs. 55%) compared to those who reported good, very good, or excellent mental health. In addition, they were more likely to report a diagnosed depression treated with medication (48% vs. 9%) and were more likely to have less than a college degree (29% vs. 15%).

Estimates of the association between component items of the REMS and poor self-rated mental health are shown in Table 2. There were several REMS items where the ORs were >1.0, but not statistically significant. For example, within the domain of “assumptions of inferiority,” for those who reported someone acting surprised at their scholastic or professional success or someone assuming they grew up in a particular neighborhood, the adjusted (Model 2) ORs compared to those who did not were 1.68 (0.59, 4.76) (68% increased risk) and 1.49 (95% CI: 0.68, 3.25) (49% increased risk), respectively. Within the domain of “second-class citizen and assumptions of criminality”, for those who reported someone avoided sitting next to them in public, or avoided eye contact with them because of their race, the adjusted ORs were 2.19 (95% CI: 0.78, 6.13) (119% increased risk) and 2.22 (95% CI: 0.81, 6.04) (122% increased risk), respectively, compared to women who reported “no”. Within the domain of microinvalidations, for those reporting that they were told not to think about race anymore or that people of all racial groups experience the same obstacles, the respective adjusted (Model 2) estimates were 1.87 (95% CI: 0.93, 3.74) and 1.83 (95% CI: 0.88, 3.81) compared to those who did not. Within the domain of exoticization, the respective multivariable-adjusted (Model 2) estimates for women who reported being told that all Asians look alike or that someone only wanted to date them because of their race, compared to those who did not, were 1.68 (95% CI: 0.82, 3.43) and 1.80 (95% CI: 0.83, 3.91). In contrast, within the domain of environmental microaggressions, observing people of the same race as CEOs of major corporations was associated with a decreased-odds of fair/poor mental health: the model 2 estimate was 0.63 (95% CI: 0.31, 1.32) (37% decreased risk) compared to those who did not. Additional adjustment for the average IM-4 score (Model 3) did not materially change the OR estimates of these REMS items with poor mental health.

A few estimates were not only elevated, but also achieved statistical significance (Table 2). Within the domain of “second-class citizen and assumptions of criminality,” for those who reported someone’s body language showed they were afraid of them, compared to those who did not, the multivariable-adjusted estimate was 4.35 (95% CI: 1.27, 14.92). Within the domain of “microinvalidations”, for women who reported being told that they should not complain about race compared to those who did not, the OR was 2.64 (95% CI: 1.27, 5.48). Finally, within the domain of “workplace and school microaggressions,” for women who reported that their opinion was overlooked in a group discussion and was ignored at school or work due to their race, the respective multivariable-adjusted estimates were 3.00 (95% CI: 1.29, 6.98) and 3.94 (95% CI: 1.56, 9.39). Further adjustment for the IM-4 score (Model 3) did not materially change any of the OR estimates.

In analyses of the IM-4 components (Table 3), we chose those who were undecided (neutral) as the reference group in order to simultaneously explore the effects of either agreement or disagreement with AA stereotypes on fair/poor self-rated mental health.

While few associations achieved statistical significance, some notable patterns were observed. For example, the number of women who agreed was consistently higher within the IM4-AO while the number who disagreed was highest within the IM4-UM domain. Within the IM4-AO domain, most estimates indicated a reduced-odds of fair/poor self-rated mental health (OR < 1.00) compared to those who were undecided. Specifically, women who agreed that AAs have stronger work ethics, are harder workers, are more motivated for success, and are more likely to be good at math and science were less likely to report poor/fair mental health compared to those who were undecided. The respective adjusted (Model 2) ORs were 0.45 (95% CI: 0.18, 1.10) (55% reduced risk), 0.59 (95% CI: 0.26, 1.35) (41% reduced risk), 0.42 (95% CI: 0.17, 1.02) (58% reduced risk), and 0.41 (95% CI: 0.13, 1.10) (59% reduced risk). Further adjustment for the REMS score (Model 3) strengthened a few estimates, elevating them to statistical significance: stronger work ethics (0.39 (95% CI: 0.15, 0.99)), motivation for success (0.32 (95% CI: 0.13, 0.81)), and good at math and science (0.36 (95% CI: 0.13, 0.99)). The corresponding estimates for those who disagreed with these statements were similar but attenuated, with the exception being those who disagreed that AAs have higher grade point averages in school because academic success is more important having an elevated OR compared to those who were undecided: 1.55 (95% CI: 0.48, 5.01) (55% increased risk). Further adjustment for the REMS score (Model 3) did not materially alter these estimates.

Within the IM4-UM domain, compared to those who were undecided, the adjusted (Model 2) estimates for those who disagreed with statements such as AAs are less likely to face barriers at work, or climb the corporate ladder more easily were 1.72 (95% CI: 0.59, 5.02) and 1.17 (95% CI: 0.49, 2.75), respectively. Additional adjustment for the REMS score (Model 3) further attenuated the respective estimates to 1.30 (0.43, 3.90) and 1.05 (0.43, 2.56). For the same variables and comparison, the adjusted ORs for those who agreed with these statements were 0.84 (95% CI: 0.23, 3.09) and 0.53 (95% CI: 0.15, 1.81), respectively. No estimates within this domain achieved statistical significance, indicating no clear evidence of an association in this sample.

We also explored the overall REMS and average IM-4 scores in relation to fair/poor self-rated mental health (Table 4). Compared to women who responded “yes” ≤5 times on the REMS, those who responded “yes” more than 10 times had significantly higher odds of reporting fair/poor mental health, with an adjusted (Model 2) OR of 2.84 (95% CI: 1.19, 6.78), and remained statistically significant after further adjustment for the IM-4 score (Model 3) (OR 2.70, 95% CI: 1.12, 6.49). In contrast, reduced associations (ORs < 1.00) were observed between the overall IM-4 average score and self-rated mental health. For example, compared to women in category 1 (low) of the IM-4 score, women in category 3 (high) had an adjusted OR of 0.60 (95% CI: 0.25, 1.41), with little change after further adjustment for the REMS score (Model 3). We further analyzed the IM-4 score according to domain (achievement orientation and upward mobility). Compared to women in category 1 (low) of the achievement orientation domain, women in category 3 (high) had an unadjusted (model 1) OR of 0.42 (95% CI:0.18, 0.96); adjustment for age and education (model 2) attenuated the OR to 0.54 (95% CI: 0.23, 1.27) but additional adjustment for the REMS score did not further impact the estimate. Further, within the upward mobility domain, models 1 and 2 did not materially differ from one another: 0.70 (0.32, 1.56) and 0.78 (95% CI: 0.34, 1.80), respectively. Adjustment for the REMS score, however, attenuated the estimate to 1.01 (95% CI: 0.41, 2.44).

Finally, we stratified according to age (≤26, >26) and questionnaire completion date (pre-pandemic, pandemic). The overall REMS score was associated with elevated risk of fair/poor mental health across both age groups, but was stronger among older women (OR = 6.00, 95% CI: 1.42, 25.39) than younger women (OR = 2.00, 95% CI: 0.67, 5.92) (Supplemental Table S1). In contrast, the average IM-4 score was associated with increased risk among the younger women (OR = 1.29, 95% CI: 0.45, 3.70) and significantly decreased risk among the older women (OR = 0.09, 95% CI: 0.01, 0.55). Finally, the risk of fair/poor mental health associated with both IM-4AO and IM-4UM, respectively, were moderately decreased and null (OR ~ 1.00) among the younger women, while both were greatly decreased among older women. Similarly, pandemic estimates were stronger in magnitude than pre-pandemic estimates for both REMS and IM-4 (Supplemental Table S2). The estimates associated with IM-4AO were decreased for both time periods, yet stronger for the pandemic group. Alternatively, the pre-pandemic IM-4UM estimates were increased (OR = 2.03, 95% CI:0.50, 8.24), while the pandemic estimates were decreased (OR = 0.61, 95%CI: 0.18, 2.09).

## 4. Discussion

In this study, we examined the relationships between experiences of racial microaggressions [15], internalization of model minority stereotypes [36], and self-rated mental health among AA women. First, several specific microaggression items were individually associated with fair/poor mental health. Second, while internalization of model minority stereotypes were generally not statistically significantly associated with self-rated mental health, a few individual items within the achievement orientation domain did show significant associations.

Third, and perhaps most notable, the frequent and cumulative experiences of microaggressions were significantly associated with fair/poor mental health. Specifically, women who reported many microaggressions (>10), had more than twice the odds of fair or poor mental health compared to those who reported fewer microaggressions (≤5). This association was statistically significant, and remained even after additional adjustment for the Internalization of the Model Minority Measure (IM-4) (Model 3). These results are consistent with the literature. Sanchez and colleagues, in a study of AA and Latinx American undergraduate students (46% female, 53% Asian), assessed the impact of microaggressions on mental health. Students who reported higher REMS scores also reported higher psychological distress, measured using the five-item Mental Health Inventory [32,58]. In another study of US university students, investigators assessed the frequency in which the students experienced over 50 types of microaggressions over the previous month and year. Students of color (26% of the study sample) reported more microaggressions than White students, and higher numbers of experiences during the previous month were associated with increased anxiety (*p* < 0.01) [31].

Among the individual REMS items, those that were both elevated and statistically significant fell within three domains: (1) “second-class citizen and assumptions of criminality” (someone’s body language showed they were scared of me because of my race), “ (2) microinvalidations” (I was told that I should not complain about race), and (3) “workplace and school microaggressions” (My opinion was overlooked in a group discussion; I was ignored at school or work because of my race). These three types of microaggressions share important commonalities: all involve being singled out, excluded, or made to feel less than others during interpersonal or professional interactions. Such experiences signal that individuals are not seen as equal members of society and can undermine their legitimacy, contributions, and sense of belonging. The association with fair and poor mental health indicates that these forms of microaggressions can have lasting effects beyond discomfort, such as impacting psychological wellbeing. This finding is supported by previous studies which reported similar attitudes towards exposures of microaggressions. For example, a 19-year old woman of Chinese descent stated: “Just like all other people of color, we are perceived as “other” in this country and, given how hard my family and I have tried to assimilate into American culture from the time I was born (in this country!!), that makes me very hurt” [23]. A focus group of AA medical students (81% female) described being viewed as “exotic”, “perpetual foreigners”, resulting in increased reports of anxiety and insecurity [33]. Finally, in a qualitative study of underrepresented health professional students (65% female, 5% AA), one student described being “invisible while standing out”, while another shared having to take medications for anxiety and depression, while being unsure whether “it’s a consequence of medical school in itself or the microaggression” [29].

With regard to the internalization of model minority stereotypes related to achievement, we found that women who agreed with statements such as, “Asian Americans have stronger work ethics” or “are more likely to be good at math and science” tended to have lower odds of reporting poor or fair mental health compared to those who were undecided. Greater internalization of the model minority stereotype may thus be associated with fewer depressive symptoms, suggesting a potential protective effect. Similar findings have been reported in a study of predominantly female AA college students (mean age = 21 years; 72% US born) where greater Internalization of the MMM, as indicated by higher IM-4 scores, were associated with fewer depressive symptoms [45]. A possible explanation, as further suggested by Keum and Wong, is that internalizing the MMM does not necessarily indicate daily behavioral adherence. Furthermore, Asian Americans may adopt “a self-protective survival strategy” by identifying with aspects of these positive stereotypes that can minimize negative self-perceptions, in turn reducing depressive symptoms [47].

Our analyses of the IM-4 scale utilized the “undecided” group as the reference category, allowing us to simultaneously explore the impact of internalization and rejection of the model minority myth. We chose to interpret “undecided” as ambivalent reflecting the feelings of confusion when appraising the stereotype. We were able to show greater agreement with items within the achievement orientation domain and greater disagreement with the items within the upward mobility domain, nuances that could otherwise be overshadowed by reliance on an overall average score. A survey from the Pew Research Center [49], asked Asian adults whether using the term “model minority” to describe Asians in the US was appropriate, included two options to capture those in the middle: “neither a good thing nor a bad thing” (28%), and “unsure” (12%). It is not possible from our data to determine whether participants selected “undecided” because they were unaware of others’ beliefs, wanted to avoid answering the question, or were truly unsure.

Previous research has also indicated that both the experiences and effects of microaggressions and the MMM are interrelated and cannot be understood in isolation [4]. We attempted to account for this by adjusting for the other score (either REMS or IM-4) in our final regression model (Model 3). Adjusting for IM-4 scores had no impacts on our REMS estimates, while adjusting for REMS scores only impacted a few of the IM-4 estimates (have stronger work ethics, are more motivated to be successful, and are more likely to be good at math and science) by increasing the magnitude of the protective association (lower ORs) and precision (smaller confidence intervals). Future research is needed to better understand the dueling impacts of microaggressions and internalizations of the MMM.

The Epi AWARE study consists only of AA women who experience distinct forms of oppression compared to AA men. Our questionnaire included two questions reflecting gendered racial microaggressions [50] which showed increased (but statistically non-significant) associations with fair/poor mental health: someone wanted to date me because of my race (74% increased risk), and someone told me that all people in my racial group look alike (64% increased risk). Thus, studying these questions in a female population may help to inform culturally appropriate mental health counseling, making mental health services more accessible and acceptable to AA women, and interrupting cycles of violence.

Epi AWARE participants range in age from 18–59 years. To explore whether our results applied to women of all ages, we performed analyses within strata of age: ≤26 vs. >26 years. The REMS was associated with elevated risk of fair/poor mental health across both age groups, but was stronger among older women, possibly representing greater cumulative exposure. Alternatively, the risk of fair/poor mental health associated with both IM-4AO and IM-4UM were greatly decreased among older women. Our results suggest that our results apply to a AA women across many decades of age, and that there may be generational differences in coping and appraisal of the MMM.

The Epi AWARE study was conducted between 2019 and 2022, during the height of the COVID-19 pandemic which could have influenced the frequency of negative encounters. Stratified analyses according to the timing of questionnaire completion showed that overall, pandemic results were slightly stronger in magnitude for both REMS and IM-4. The pre-pandemic REMS, however was still elevated indicating a 91% increased risk of fair/poor mental health for those scoring highest category compared to the lowest supporting the evidence of the long pre-existence of anti-AA racism.

Our study has several limitations, the first being its cross-sectional design. Both exposures (REMS, IM-4) and the outcome under study (self-rated mental health) were measured simultaneously, thus, we could not establish the temporal sequence between the two. The REMS is designed to capture experiences within the past six months [15], while the IM-4 has demonstrated reliability and consistency over a two-week period [36]. It is also possible that one’s self-reported mental health may have impacted the appraisal of interpersonal encounters and acceptance or rejection of the MMM. Second, our analytic sample was limited (n = 152) and influenced analytic descisions and the precision of our estimates. For example, we were unable to perform analyses according to participant ethnicity. Research suggests that both stereotypes (e.g., perpetual foreigner) and acts of discrimination towards AAs vary depending on ethnicity. For example, Goh and McCue [59] found that Americans percieved East Asians as being least “foreign”, followed by Southeast Asians, South Asians, and West Asians. In one study, South Asians were more likely to report workplace discrimination [60], while in another, West Asians (encompassing Arab and Middle Eastern countries) were considered the most foreign and inferior [61], and frequently stereotyped as dangerous (i.e., terrorists) [59]. Future studies which disaggregate Asian subgroups are needed to better understand and address ethnicity-specific barriers to education, employment, and ultimately, somatic and mental health.

We also dichotomized our outcome variable largely due to sparse data concerns. In our analysis, only 11 (7%) women reported “poor” mental health, while 48 women reported “fair”; thus, we combined the fair and poor categories. A recent analysis of Canadian self-rated health using the same 5-point scale found that using lower cut points (i.e., fair/poor vs. excellent/very good/good) may improve sensitivity to outcome variability [62].

Few of our estimates achieved statistical significance despite estimates of reasonable magnitude. It is possible that these findings resulted from sample size limitations, the complex nature of the MMM, in particular, and the design of the IM-4 itself. While some of the estimates for individual components of the REMS achieved statistical significance, the confidence intervals were wide indicating low precision. Further, cell sizes for many of the subgroups in the IM-4 analyses (e.g., the undecided subgroups) were quite small (e.g., ≤30 respondents), resulting in lower statistical precision.

Finally, we did not assess how participants cope with stress and adversity. Individual appraisal and subsequent coping methods in response to an event influences the degree of psychological stress experienced after an event [63]. Blume et al. found that self-efficacy to cope was a potential modifiable variable in the relationship between microaggressions and anxiety [31].

Our study also has several notable strengths. First, we utilized established instruments to assess microaggressions and internalization of the MMM. The REMS has been shown to be a reliable measure of discrimination across four major racial groups, specifically, Asian Americans, Latina/o Americans, Black/African Americans, and multiracial people [15]. The IM-4 [36] with its two-factor structure, measures the unique racialized experiences of Asian Americans, and has demonstrated internal reliability and stability over a 2- week period. We did not, however, utilize the full set of items for either the REMS or IM-4 instruments. Our selection of items was based on considerations of Epi AWARE questionnaire design and length, and not on any a priori hypotheses or strategy. Nonetheless, we did select questions across all domains of the REMS and IM-4, and attempted to retain those we felt were least repetitive and would be most relevant to our potential participants. It is possible that our selection of items did not adequately represent the full range of experiences of the women in our cohort. It is also possible that our selection of a subset of scale items compromised the performance properties (i.e., validity and reliability) of these measures. Our Cronbach’s alpha coefficients, however, indicate acceptable reliability. Finally, we used a single-item to measure self-assessed mental health. This single-item measure was found to be associated with mental health measures including the Center for Epidemiologic Studies Depression Scale (CES-D), Geriatric Depression Scale-Short Form (GDS-SF), and Patient Health Questionnaire-9 (PHQ-9) [53]. Further, self-rated mental health has been shown to be significantly associated with diagnoses for any 12-month DSM-IV psychiatric disorder in specific Asian American populations (e.g., Filipinos). As such, this measure is increasingly employed in epidemiological research and serves as a valuable complement to clinician-administered mental health assessments [58].

## 5. Conclusions

In conclusion, experiencing frequent microaggressions (particularly those related to assumptions of criminality, invalidation, or workplace exclusion) was significantly and consistently associated with fair/poor mental health within this cohort of Asian American women. In addition, endorsement of Asians’ achievement stereotypes was generally associated with better self-rated mental health, possibly the result of “a self-protective survival strategy” within this population. A greater understanding and acknowledgement of the impacts of subtle microaggressions and stereotyping, including the internalization of stereotypes, is necessary in order to accurately support psychological wellbeing and design effective mental health interventions for Asian American women.

## Figures and Tables

**Table 1:** Characteristics of study participants overall and according to Self-rated Mental Health, Epi AWARE (n = 152).

		Self-Rated Mental Health	
Characteristics	Total Study Sample	Good/ Very Good/ Excellent	Fair/Poor
	n = 152	n = 93	n = 59
Age (years), mean (SD)	28.7 (9.3)	30.2 (9.7)	26.3 (8.3)
Education (years), n (%)			
≤15	31 (20.4)	14 (15.1)	17 (28.8)
16	51 (33.6)	27 (29.0)	24 (40.7)
≥17	70 (46.0)	52 (55.9)	18 (30.5)
Ethnic Geography, n (%)			
South Asian	12 (7.9)	7 (7.5)	5 (8.5)
East Asian	110 (72.4)	68 (73.1)	42 (71.2)
South East Asian	30 (19.7)	18 (19.4)	12 (20.3)
Nativity, n (%)			
U.S. Born	98 (64.5)	51 (54.8)	47 (79.7)
Depression Treated with Medication, n (%)			
Yes	36 (23.7)	8 (8.6)	28 (47.5)

**Table 2:** Odds Ratios (OR) and 95% Confidence Intervals (95% CI) for Fair/Poor Self-Rated Mental Health from Epi AWARE Study (n = 152).

			Fair/Poor Self-Rated Health OR (95% CI)		
Racial and Ethnic Microaggression Scale (REMS)		n	Model 1	Model 2	Model 3
***Assumptions of Inferiority***					
Someone assumed that I was poor because of my race.	No	136	1.00 (Reference)	1.00 (Reference)	1.00 (Reference)
	Yes	16	1.67 (0.59, 4.71)	1.39 (0.47, 4.13)	1.52 (0.50, 4.59)
Someone acted surprised at my scholastic or professional success because of my race.	No	134	1.00 (Reference)	1.00 (Reference)	1.00 (Reference)
	Yes	18	1.68 (0.63, 4.51)	1.68 (0.59, 4.76)	1.72 (0.60, 4.96)
Someone assumed I grew up in a particular neighborhood because of my race.	No	114	1.00 (Reference)	1.00 (Reference)	1.00 (Reference)
	Yes	38	1.60 (0.76, 3.37)	1.49 (0.68, 3.25)	1.52 (0.69, 3.30)
Someone told me that I was “articulate” after she/he assumed I wouldn’t be.	No	113	1.00 (Reference)	1.00 (Reference)	1.00 (Reference)
	Yes	39	1.13 (0.54, 2.38)	1.10 (0.51, 2.41)	1.05 (0.48, 2.30)
***Second-class citizen/*** ***Assumption of Criminality***					
Someone clenched his/her purse or wallet upon seeing me because of my race	No	149	1.00 (Reference)	1.00 (Reference)	1.00 (Reference)
	Yes	3	---* (---, ---) *	---* (---, ---) *	---* (---, ---) *
Someone avoided sitting next to me in a public space (e.g., restaurants, subways) because of my race.	No	133	1.00 (Reference)	1.00 (Reference)	1.00 (Reference)
	Yes	19	2.44 (0.92, 6.47)	2.19 (0.78, 6.13)	2.20 (0.78, 6.16)
Someone avoided eye contact with me because of my race.	No	132	1.00 (Reference)	1.00 (Reference)	1.00 (Reference)
	Yes	20	2.14 (0.83, 5.53)	2.22 (0.81, 6.04)	2.31 (0.84, 6.33)
Someone’s body language showed they were scared of me because of my race	No	138	1.00 (Reference)	1.00 (Reference)	1.00 (Reference)
	Yes	14	3.17 (1.01, 9.97) **	4.35 (1.27,14.92) **	4.25 (1.25,14.51) **
I received substandard service in stores compared to customers of other racial groups.	No	96	1.00 (Reference)	1.00 (Reference)	1.00 (Reference)
	Yes	56	1.47 (0.75, 2.88)	1.45 (0.72, 2.95)	1.46 (0.72, 2.96)
***Microinvalidations***					
Someone told me that people should not think about race anymore.	No	78	1.00 (Reference)	1.00 (Reference)	1.00 (Reference)
	Yes	74	1.80 (0.93, 3.49)	1.87 (0.93, 3.74)	1.80 (0.89, 3.63)
Someone told me that she or he was color-blind.	No	91	1.00 (Reference)	1.00 (Reference)	1.00 (Reference)
	Yes	61	1.46 (0.75, 2.84)	1.43 (0.71, 2.89)	1.35 (0.66, 2.76)
I was told that people of color do not experience racism anymore.	No	88	1.00 (Reference)	1.00 (Reference)	1.00 (Reference)
	Yes	64	2.26 (1.16, 4.40) **	1.85 (0.92, 3.72)	1.74 (0.85, 3.55)
Someone of a different racial group has stated that there is no difference between the two of us.	No	96	1.00 (Reference)	1.00 (Reference)	1.00 (Reference)
	Yes	56	1.86 (0.95, 3.66)	1.79 (0.88, 3.62)	1.74 (0.86, 3.54)
I was told that I should not complain about race.	No	102	1.00 (Reference)	1.00 (Reference)	1.00 (Reference)
	Yes	50	2.92 (1.45, 5.87) **	2.64 (1.27, 5.48) **	2.54 (1.20, 5.33) **
I was told that people of all racial groups experience the same obstacles.	No	105	1.00 (Reference)	1.00 (Reference)	1.00 (Reference)
	Yes	47	2.09 (1.04, 4.21) **	1.83 (0.88, 3.81)	1.79 (0.85, 3.77)
***Exoticization/Assumptions of Similarity***					
Someone assumed that I spoke a language other than English.	No	29	1.00 (Reference)	1.00 (Reference)	1.00 (Reference)
	Yes	123	1.52 (0.64, 3.62)	1.26 (0.51, 3.14)	1.25 (0.50, 3.13)
Someone assumed that I ate foods associated with my race/culture every day.	No	72	1.00 (Reference)	1.00 (Reference)	1.00 (Reference)
	Yes	80	1.56 (0.80, 3.01)	1.51 (0.76, 3.03)	1.52 (0.75, 3.10)
Someone told me that all people in my racial group look alike.	No	65	1.00 (Reference)	1.00 (Reference)	1.00 (Reference)
	Yes	87	2.06 (1.04, 4.07) **	1.68 (0.82, 3.43)	1.64 (0.80, 3.38)
Someone wanted to date me only because of my race	No	112	1.00 (Reference)	1.00 (Reference)	1.00 (Reference)
	Yes	40	1.63 (0.78, 3.38)	1.80 (0.83, 3.91)	1.74 (0.80, 3.81)
***Environmental*** ***Microaggressions***					
I observed people of my race portrayed positively in movies	No	59	1.00 (Reference)	1.00 (Reference)	1.00 (Reference)
	Yes	93	0.99 (0.51, 1.93)	0.90 (0.44, 1.85)	0.91 (0.44, 1.87)
I observed that people of my race were the CEOs of major corporations	No	100	1.00 (Reference)	1.00 (Reference)	1.00 (Reference)
	Yes	52	0.59 (0.29, 1.19)	0.63 (0.30, 1.32)	0.65 (0.31, 1.36)
I observed that someone of my race is a government official in my state.	No	94	1.00 (Reference)	1.00 (Reference)	1.00 (Reference)
	Yes	58	1.19 (0.61, 2.32)	1.29 (0.64, 2.62)	1.26 (0.62, 2.56)
***Workplace and School*** ***Microaggressions***					
An employer or co-worker was unfriendly or unwelcoming toward me because of my race.	No	121	1.00 (Reference)	1.00 (Reference)	1.00 (Reference)
	Yes	31	1.64 (0.74, 3.64)	1.40 (0.61, 3.21)	1.36 (0.59, 3.13)
My opinion was overlooked in a group discussion because of my race	No	116	1.00 (Reference)	1.00 (Reference)	1.00 (Reference)
	Yes	36	2.12 (0.99, 4.53) **	3.00 (1.29, 6.98) **	2.87 (1.22, 6.77) **
I was ignored at school or at work because of my race.	No	119	1.00 (Reference)	1.00 (Reference)	1.00 (Reference)
	Yes	33	3.16 (1.42, 6.99) **	3.94 (1.66, 9.39) **	3.78 (1.56, 9.18) **
An employer or co-worker treated me differently than White co-workers.	No	94	1.00 (Reference)	1.00 (Reference)	1.00 (Reference)
	Yes	58	1.50 (0.77, 2.93)	1.70 (0.83, 3.45)	1.65 (0.81, 3.37)

Model 1: unadjusted. Model 2: adjusted for age (years) and education (years). Model 3: adjusted for Model 2 variables plus IM-4 score. * model did not converge given sparse sample size. ** significant at the α = 0.05 threshold.

**Table 3:** Odds ratios and 95% Confidence Intervals (CI) for IM-4 and self-rated mental health (poor/fair), Epi AWARE Study (n = 152).

		Odds Ratio (95% Confidence Interval)		
Asian Americans:	n	Model 1	Model 2	Model 3
*Achievement Orientation*				
have stronger work ethics				
Undecided	31	1.00 (reference)	1.00 (reference)	1.00 (reference)
Agree	79	0.43 (0.19, 1.02)	0.45 (0.18, 1.10)	0.39 (0.15, 0.99) *
Disagree	42	0.70 (0.28, 1.79)	0.54 (0.20, 1.46)	0.42 (0.15, 1.20)
are harder workers				
Undecided	39	1.00 (reference)	1.00 (reference)	1.00 (reference)
Agree	78	0.58 (0.27, 1.28)	0.59 (0.26, 1.35)	0.53 (0.22, 1.23)
Disagree	35	0.88 (0.35, 2.19)	0.69 (0.26, 1.84)	0.50 (0.21, 1.56)
are more likely to achieve academic and economic success				
Undecided	29	1.00 (reference)	1.00 (reference)	1.00 (reference)
Agree	97	0.70 (0.30, 1.61)	0.89 (0.37, 2.15)	0.75 (0.30, 1.86)
Disagree	26	0.90 (0.31, 2.63)	0.90 (0.30, 2.75)	0.64 (0.20, 2.07)
are more motivated to be successful				
Undecided	30	1.00 (reference)	1.00 (reference)	1.00 (reference)
Agree	92	0.42 (0.18, 0.98) *	0.42 (0.17, 1.02)	0.32 (0.13, 0.81) *
Disagree	30	0.67 (0.24, 1.85)	0.49 (0.17, 1.45)	0.30 (0.09, 0.96) *
generally have higher grade point averages in school because academic success is more important				
Undecided	23	1.00 (reference)	1.00 (reference)	1.00 (reference)
Agree	103	0.64 (0.26, 1.61)	0.73 (0.28, 1.92)	0.73 (0.27, 1.96)
Disagree	26	1.77 (0.57, 5.51)	1.55 (0.48, 5.01)	1.47 (0.44, 4.92)
get better grades in school because they study harder				
Undecided	26	1.00 (reference)	1.00 (reference)	1.00 (reference)
Agree	91	0.55 (0.22, 1.33)	0.59 (0.23, 1.50)	0.56 (0.22, 1.46)
Disagree	35	1.24 (0.45, 3.42)	1.05 (0.36, 3.05)	0.89 (0.29, 2.67)
are more likely to be good at math and science				
Undecided	34	1.00 (reference)	1.00 (reference)	1.00 (reference)
Agree	44	0.47 (0.19, 1.20)	0.41 (0.15, 1.10)	0.36 (0.13, 0.99) *
Disagree	74	0.77 (0.34, 1.74)	0.62 (0.26, 1.50)	0.54 (0.22, 1.34)
*Upward Mobility*				
are less likely to face barriers at work				
Undecided	23	1.00 (reference)	1.00 (reference)	1.00 (reference)
Agree	28	1.13 (0.33, 3.92)	0.84 (0.23, 3.09)	0.65 (0.17, 2.51)
Disagree	101	2.27 (0.83, 6.25)	1.72 (0.59, 5.02)	1.30 (0.43, 3.90)
are less likely to experience racism in the United States				
Undecided	22	1.00 (reference)	1.00 (reference)	1.00 (reference)
Agree	17	0.96 (0.26, 3.58)	0.75 (0.19, 3.05)	0.78 (0.19, 3.23)
Disagree	113	1.16 (0.45, 2.99)	0.90 (0.33, 2.46)	0.67 (0.23, 1.89)
are more likely to be treated as equals to European Americans				
Undecided	20	1.00 (reference)	1.00 (reference)	1.00 (reference)
Agree	25	1.24 (0.37, 4.19)	1.01 (0.28, 3.66)	1.07 (0.29, 4.00)
Disagree	107	1.20 (0.44, 3.25)	0.91 (0.31, 2.62)	0.86 (0.29, 2.56)
climb the corporate ladder more easily				
Undecided	35	1.00 (reference)	1.00 (reference)	1.00 (reference)
Agree	22	0.72 (0.22, 2.32)	0.53 (0.15, 1.81)	0.41 (0.11, 1.53)
Disagree	95	1.46 (0.65, 3.26)	1.17 (0.49, 2.75)	1.05 (0.43, 2.56)

Model 1: unadjusted. Model 2: adjusted for age (years) and education (years). Model 3: adjusted for Model 2 variables plus REMS score. * Statistically significant at the α = 0.05 threshold.

**Table 4:** Odds ratios and 95% CI of Racial and Ethnic Microaggressions Scale (REMS) and Internalization of the Model Minority Myth (IM-4) Scale Score and Self-Rated Mental Health (fair/poor), Epi AWARE Study (n = 152). 1, 2 and 3 refer to the REMS and IM-4 categories from the lowest to highest.

			Odds Ratio (95% Confidence Interval)					
	Total	Cases	Model 1		Model 2		Model 3 ^1,2^	
REMS (Number of Times Responded “yes”)								
1 (Low)	53	15	1.00	Reference	1.00	Reference	1.00	Reference
2	52	18	1.34	(0.59, 3.07)	1.13	(0.47, 2.69)	1.08	(0.45, 2.60)
3 (High)	47	26	3.14	(1.37, 7.19) *	2.84	(1.19, 6.78) *	2.70	(1.12, 6.49) *
IM-4 (average score, categories)								
1 (Low)	42	21	1.00	Reference	1.00	Reference	1.00	Reference
2	53	20	0.61	(0.27, 1.38)	0.69	(0.30, 1.64)	0.73	(0.31, 1.77)
3 (High)	57	18	0.46	(0.20, 1.05)	0.60	(0.25, 1.41)	0.66	(0.27, 1.61)
IM-4: Achievement Orientation (average score, categories)								
1 (Low)	40	22	1.00	Reference	1.00	Reference	1.00	Reference
2	53	17	0.39	(0.17, 0.90) *	0.46	(0.19, 1.11)	0.53	(0.21, 1.30)
3 (High)	59	20	0.42	(0.18, 0.96) *	0.54	(0.23, 1.27)	0.59	(0.24, 1.42)
IM-4: Upward Mobility (average score, categories)								
1 (Low)	46	20	1.00	Reference	1.00	Reference	1.00	Reference
2	49	19	0.82	(0.36, 1.87)	0.94	(0.40, 2.21)	1.06	(0.44, 2.57)
3 (High)	57	20	0.70	(0.32, 1.56)	0.78	(0.34, 1.80)	1.01	(0.41, 2.44)

Model 1: unadjusted. Model 2: adjusted for age (years) and education (years). ^1^ Model 3: adjusted for Model 2 variables plus IM-4 score. ^2^ Model 3: adjusted for Model 2 variables plus REMS score. * significant at the α = 0.05 threshold.

## Data Availability

Data underlying the study cannot be made publicly available due to ethical concerns about patient confidentiality. Data will be made available to qualified researchers on request to epiaware@bu.edu.

## Supplementary Materials

The following supporting information can be downloaded at: https://www.mdpi.com/article/10.3390/ijerph22121869/s1, Table S1: Odds ratios and 95% confidence intervals of Racial and Ethnic Microaggressions Scale (REMS) and Internalization of the Model Minority Myth (IM-4) Scale and Self-Rated Mental Health (fair/poor), according to participant age (≤26 vs >26 years), Epi AWARE Study; Table S2: Odds ratios and 95% confidence intervals of Racial and Ethnic Microaggressions Scale (REMS) and Internalization of the Model Minority Myth (IM-4) Scale and Self-Rated Mental Health (fair/poor), according to timing of questionnaire completion (pre-COVID-19 pandemic v during the COVID-19 pandemic), Epi AWARE Study.

## Abbreviations

The following abbreviations are used in this manuscript:

US	United States
AA	Asian American
REMS	Racial and Ethnic Microaggressions Scale
IM-4	Internalization of the Model Minority Myth Measure
IM4-AO	Internalization of the Model Minority Myth Measure—Achievement Orientation
IM4-UM	Internalization of the Model Minority Myth Measure—Upward Mobility
OR	Odds Ratio
CI	Confidence Interval
