# Supplementary material for: The Association Between Racial Microaggressions and Stereotypes and Self-Rated Mental Health in Asian Women
Source: Int J Environ Res Public Health. 2025 Dec 15;22(12):1869. doi: 10.3390/ijerph22121869 (PMC12732802; doi:10.3390/ijerph22121869)
Supplement: Supplementary file 1 [file ijerph-22-01869-s001.zip › ijerph-3957709-supplementary.pdf]

Supplemental Table S1. Odds ratios and 95% confidence intervals of Racial and Ethnic Microaggressions Scale (REMS) and Internalization of the Model Minority Myth (IM-4) Scale and Self-Rated Mental Health (fair/poor), according to participant age (≤26 vs >26 years), Epi AWARE Study

	OR (95% CI)							
	≤ 26 years (N=75)				>26 years (N=77)			
	Model 1		Model 2		Model 1		Model 2	
**REMS (number of times responded "yes")**								
**1** (Low)	1.00	Reference	1.00	Reference	1.00	Reference	1.00	Reference
**2**	1.67	(0.50, 5.51)	1.53	(0.43, 5.40)	1.06	(0.31, 3.66)	0.93	(0.26, 3.37)
**3** (High)	1.85	(0.50, 6.83)	1.91	(0.48, 7.63)	4.51	(1.38, 14.82)*	3.50	(1.00, 12.22)*
**IM-4 (average score, categories)**								
**1** (Low)	1.00	Reference	1.00	Reference	1.00	Reference	1.00	Reference
**2**	0.76	(0.20, 2.93)	0.73	(0.18, 3.00)	1.02	(0.32, 3.24)	1.53	(0.42, 5.55)
**3** (High)	1.05	(0.28, 3.94)	1.45	(0.35, 5.96)	0.33	(0.10, 1.08)	0.41	(0.12, 1.44)
**IM-4: Achievement Orientation (average score, categories)**								
**1** (Low)	1.00	Reference	1.00	Reference	1.00	Reference	1.00	Reference
**2**	0.18	(0.04, 0.77)*	0.15	(0.03, 0.70)*	0.69	(0.22, 2.23)	1.15	(0.31, 4.24)
**3** (High)	0.55	(0.16, 1.87)	0.72	(0.19, 2.74)	0.35	(0.11, 1.18)	0.45	(0.12, 1.59)
**IM-4: Upward Mobility (average score, categories)**								
**1** (Low)	1.00	Reference	1.00	Reference	1.00	Reference	1.00	Reference
**2**	1.56	(0.39, 6.24)	1.50	(0.35, 6.41)	0.85	(0.27, 2.63)	1.13	(0.33, 3.84)
**3** (High)	2.14	(0.56, 8.17)	2.03	(0.50, 8.24)	0.49	(0.15, 1.58)	0.61	(0.18, 2.09)

Model 1: unadjusted

Model 2: adjusted for age (years) and education (years).

*significant at the $\alpha = 0.05$ threshold

Supplemental Table S2. Odds ratios and 95% confidence intervals of Racial and Ethnic Microaggressions Scale (REMS) and Internalization of the Model Minority Myth (IM-4) Scale and Self-Rated Mental Health (fair/poor), according to timing of questionnaire completion (pre-COVID-19 pandemic v during the COVID-19 pandemic), Epi AWARE Study.

	OR (95% CI)							
	Pre-pandemic (N= 88)				Pandemic (N= 64)			
	Model 1		Model 2		Model 1		Model 2	
**REMS (number of times responded "yes")**								
**1** (Low)	1.00	Reference	1.00	Reference	1.00	Reference	1.00	Reference
**2**	1.20	(0.42, 3.40)	1.23	(0.43, 3.52)	0.71	(0.12, 4.30)	0.71	(0.12, 4.30)
**3** (High)	1.91	(0.65, 5.60)	2.00	(0.67, 5.92)	5.33	(1.28, 22.15)*	6.00	(1.42, 25.39)*
**IM-4 (average score, categories)**								
**1** (Low)	1.00	Reference	1.00	Reference	1.00	Reference	1.00	Reference
**2**	0.63	(0.23, 1.74)	0.64	(0.23, 1.77)	0.65	(0.15, 2.80)	0.56	(0.13, 2.48)
**3** (High)	1.27	(0.45, 3.64)	1.29	(0.45, 3.70)	0.10	(0.02, 0.63)*	0.09	(0.01, 0.55)*
**IM-4: Achievement Orientation (average score, categories)**								
**1** (Low)	1.00	Reference	1.00	Reference	1.00	Reference	1.00	Reference
**2**	0.41	(0.14, 1.16)	0.40	(0.14, 1.14)	0.50	(0.10, 2.40)	0.42	(0.08, 2.08)
**3** (High)	0.82	(0.29, 2.31)	0.80	(0.28, 2.25)	0.23	(0.05, 1.20)	0.19	(0.04, 1.04)
**IM-4: Upward Mobility (average score, categories)**								
**1** (Low)	1.00	Reference	1.00	Reference	1.00	Reference	1.00	Reference
**2**	0.70	(0.25, 1.99)	0.71	(0.25, 2.02)	1.40	(0.33, 5.93)	1.28	(0.30, 5.49)
**3** (High)	1.06	(0.39, 2.91)	1.05	(0.38, 2.87)	0.39	(0.08, 2.04)	0.36	(0.07, 1.89)

Model 1: unadjusted

Model 2: adjusted for age (years) and education (years).

*Significant at the $\alpha = 0.05$ threshold
